# Representational momentum in vision and touch: Visual motion information biases tactile spatial localization

**DOI:** 10.3758/s13414-020-01989-1

**Published:** 2020-03-05

**Authors:** Simon Merz, Hauke S. Meyerhoff, Christian Frings, Charles Spence

**Affiliations:** 1 Department of Psychology, Cognitive Psychology, University of Trier, Universitätsring 15, 54286 Trier, Germany; 2 Leibniz-Institut für Wissensmedien, Tübingen, Germany; 3 Department of Experimental Psychology, University of Oxford, Oxford, UK

**Keywords:** Crossmodal dynamic capture, Crossmodal perception, Localization, Vision, Touch, Representational momentum

## Abstract

After an object disappears, the vanishing point is shifted in the direction of motion, a phenomenon known as *representational momentum*. The present study focused on the relationship between motion information and spatial location in a crossmodal setting. In two visuotactile experiments, we studied how motion information in one sensory modality affects the perceived final location of a motion signal (congruent vs. incongruent left-right motion direction) in another modality. The results revealed a unidirectional crossmodal influence of motion information on spatial localization performance. While visual motion information influenced the perceived final location of the tactile stimulus, tactile motion information had no influence on visual localization. These results therefore extend the existing literature on crossmodal influences on spatial location and are discussed in relation to current theories of multisensory perception.

## Introduction

One of the most important questions in human perception research is to determine which information is used to inform perception, given that we live in a dynamic, multisensory world (e.g., Calvert, Spence, & Stein, 2004). In order to understand how we perceive and process the stimulation from different senses, researchers have identified and investigated systematic perceptual biases and effects in different sensory modalities. One of these systematic biases is the so-called forward displacement, that is, a dynamic visual object will be systematically misperceived further along its anticipated trajectory than its actual final location (*representational momentum*, Freyd & Finke, 1984; see Hubbard, 2005, 2018, for reviews). This bias, in which the direction of a dynamic stimulus influences its perceived spatial location, has been evidenced in vision (e.g., Freyd & Finke, 1984), audition (e.g., Feinkohl, Locke, Leung, & Carlile, 2014; Getzmann & Lewald, 2007), and touch (Merz, Deller, Meyerhoff, Spence, & Frings, 2019; Merz, Meyerhoff, Spence, & Frings, 2019). As of yet, however, crossmodal studies are sparse (though for one audio-visual study, see Hubbard & Courtney, 2010; see also Teramoto, Hidaka, Gyoba, & Suzuki, 2010). In fact, crossmodal effects on the perceived location (*spatial ventriloquism effect:* e.g., Alais & Burr, 2004; Bertelson, 1998; Caclin, Soto-Faraco, Kingstone, & Spence, 2002; Chen & Vroomen, 2013; Jackson, 1953; Pick, Warren, & Hay, 1969) and motion direction (*crossmodal dynamic capture:* e.g., Craig, 2006; Lyons, Sanabria, Vatakis, & Spence, 2006; Occelli, Spence, & Zampini, 2009; Soto-Faraco, Kingstone, & Spence, 2003; Soto-Faraco, Spence, & Kingstone, 2004a, 2004b) have been investigated in isolation, but never combined. While it is often argued that spatial co-location is a crucial factor facilitating multisensory integration, it turns out that this is typically only true when the participant’s task is, in some sense, spatial (see Spence, 2013, for a review).

In the present visuo-tactile study, the focus was on the crossmodal impact of motion information in one modality on perceived spatial location in another (see Whitney, 2002, for a detailed discussion concerning the interactions between the separate intramodal processing of motion and location, see also Hubbard, 1993). We test this by combining the logic of classical crossmodal dynamic capture studies (e.g., Craig, 2006; Lyons et al., 2006) with our recently developed representational momentum paradigm (Merz, Deller, et al., 2019; Merz, Meyerhoff, et al., 2019). In the latter paradigm, a sequence of three adjacently presented vibrotactile stimuli were presented to the left forearm (see also Merz, Deller, et al., 2019; Merz, Meyerhoff, et al., 2019). At the same time, a sequence of three adjacent visual squares was presented to indicate an implied motion sequence in a specific direction (e.g., Hubbard, 2005, 2018). The participants had to indicate the perceived location of the final, third vibrotactile stimulus / visual square. Using this experimental set-up, the direction of the visual and tactile implied motion sequence was either congruent, i.e., in the same left-to-right or right-to-left direction, or else it was incongruent (see Kerzel, 2004, for the effect of attentional load, that is, attending to multiple or only a single trajectory on the perception of moving stimuli). The participants had to judge the final location of either the visual or the tactile stimulus, therefore the directional feature information of the stimulus was not directly related to the execution of the participant’s localization task.

For the influence of motion on spatial localization in the task-relevant modality, we expect a forward displacement in motion direction for the visual modality (Freyd & Finke, 1984; for reviews, see Hubbard, 2005, 2018). That is, the final location of the sequence is misperceived in the direction of motion. With regard to the tactile modality, the previous evidence is mixed, and hence there is no clear prediction as to whether the perceived location is perceived ahead in motion direction (forward displacement: Merz, Deller, et al., 2019; Merz, Meyerhoff, et al., 2019) or whether instead it lags behind (backward displacement: Macauda, Lenggenhager, Meier, Essick, & Brugger, 2018). Importantly, however, we are interested in whether the motion information in the task-irrelevant modality affects the perception of final location (i.e., the existence of overall forward or backward displacement is irrelevant). Given the more accurate localization performance of the visual modality in the foveal region (Pick et al., 1969; Sheth & Shimojo, 2001; Wässle, Grünert, Röhrenbeck, & Boycott, 1990) compared to tactile sensory acuity at the forearm (Gallace & Spence, 2014; Weinstein, 1968), the directional information of the visual stimulus should be more accurate than the tactile directional information. Subsequently, we predict that a crossmodal influence of the visual motion on the perceived location in the tactile modality is very likely (Posner, Nissen, & Klein, 1976). Regarding the impact of tactile motion sequences on visual localization performance, it remains an exploratory research question as to whether this impact is weaker or perhaps even absent altogether.

## Experiment 1

### Methods

#### Participants

Visual and tactile displacement on their own typically elicit medium to large effect sizes (dz around 0.7), therefore we aimed for at least 19 participants to find the unisensory displacement at the minimum (α < .05; 1-β > .90; power analyses were run with G-Power 3.1.9.2, option ‘means: difference from constant’; Faul, Erdfelder, Buchner, & Lang, 2009). To account for possible dropouts, 24 participants from the University of Trier took part in this study. The data from two of the participants were excluded, one because he/she indicated the same location on more than 120 consecutive trials, the other because he/she only indicated the location of the tactile stimulus, and never the location of the visual stimulus, throughout the entire experiment. The remaining 22 participants (three male, mean age, 23.4 years, 19–30 years old, two left-handed) reported normal or corrected-to-normal vision and no sensory impairment on their forearms.

#### Apparatus and stimuli

**Fig. 1.**
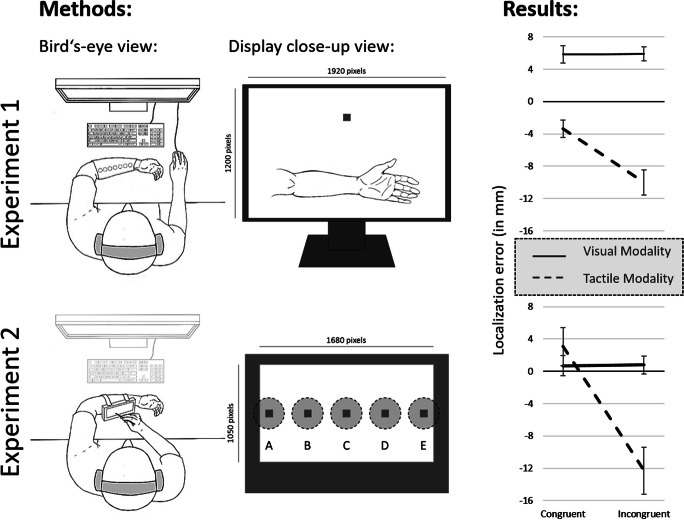
Bird’s-eye, display close-up view, and results for Experiments 1 and 2. Methods: In Experiment 1, the visual stimuli as well as the drawing of the forearm used to indicate the corresponding visual and tactile location were presented on the computer screen. The visual stimuli (*dark grey squares*) were presented as well as estimated on the upper half of the screen, the tactile stimuli were presented on the participant’s forearm, and estimated on the lower half of the screen. In Experiment 2, the tactors were attached to the back of the tablet (see dashed, *light grey circles*), and at approximately the corresponding locations, the visual stimuli (*dark grey squares*) were presented. Results: Mean localization errors as a function of directional congruency (congruent vs. incongruent) and target modality (visual vs. tactile) in Experiments 1 and 2. *Error bars* represent the standard errors following Morey (2008)

Each participant was tested individually in a dark, sound-attenuated room. The participant’s left forearm was stationary and oriented parallel to the screen (see Fig. 1) throughout the entire experiment. During the experiment, the participants wore a custom-made arm bandage with seven tactors (Model C-2, Engineering Acoustic, Inc.; controlled via the serial interface) on the interior surface. Participants used their right hand to operate the computer mouse (connected via USB). For this study, only the inner five tactors were used. The tactors (3 cm in diameter; 0.79 cm thick; centrally located skin contactor of 0.76 cm) were ordered in a straight line with an approximate center-to-center distance of 3.5 cm. The tactile stimuli (~ 250 Hz, about 200 μm peak-to-peak amplitude) were applied to the volar (inner) side of the left forearm (see also Fig. 1). The participants wore earplugs (noise reduction: 29 dB) on top of which brown noise was presented over headphones (over-ear headphones: ~ 85 dB).

Visual stimuli were presented on a 24-in. TFT screen (1920 x 1200 pixels, pixel-per-cm (PPcm): 37)*.* All of the visual stimuli were presented against a dark grey (RGB value: 192, 192, 192) background, the visual stimulus was a blue (RGB value: 0, 0, 255) square (30 x 30 pixels; 0.81 x 0.81 cm). The squares were presented in the upper half of the screen. A drawing of a left forearm, presented in white (RGB value: 0, 0, 0) with a black outline (RGB value: 255, 255, 255) on the dark grey background, was presented on the lower half of the screen for participants to mark the location of the final tactile location (see Fig. 1). The size of the drawing of the forearm from the elbow joint to the wrist was fixed for all participants at about 20.5 cm. To indicate the modality that was relevant for the response, either the upper (to respond to the visual modality) or lower (to respond to the tactile modality) half of the screen was surrounded by a 20-pixel (0.54 cm) wide green (RGB value: 0, 128, 0) rectangle. The experiment was run with E-Prime 2.0, statistical analyses were conducted with SPSS (Version 26).

#### Procedure

The experiment started with a practice phase of 12 trials, followed by a baseline block (60 trials), a motion block (320 trials), and finally a second baseline block (60 trials). All baseline data were collapsed across the first and second block, as the estimations did not vary between blocks (for more details, see the Appendix). In trials of the baseline blocks, only one visual and one tactile stimulus was presented simultaneously, to measure the localization accuracy of the participant in the current experimental setup. In between the baseline blocks, the motion block was presented. In trials of the motion block, sequences of three visual and tactile stimuli were presented simultaneously in each modality.

Each trial started with the visual presentation of a plus sign for 400 ms from the center of the screen. Thereafter, for the baseline blocks, one vibrotactile stimulus as well as one visual square were presented simultaneously with a duration of 250 ms. For the motion block, a sequence of three vibrotactile stimuli was presented successively for 250 ms each at an interstimulus interval of 250 ms. Temporally coincident with the sequence of vibrotactile stimuli, the sequence of three visual squares was presented. Following the offset of the last vibration / square, the response display was presented. The drawing of a left forearm was displayed on the lower half of the screen. Furthermore, the green rectangle, surrounding either the upper or lower half of the screen, was presented. The participants had to move a crosshair, which appeared at the center of the screen, with the help of the mouse to the corresponding location and click on the last location of the corresponding stimulus. The participants were not told to indicate a specific part of the stimulus (which might have introduced noise to the data, see Hubbard & Ruppel, 2018). Importantly, for the tactile stimulus, the participants had to transform the perceived final location on the forearm to the drawing of the forearm presented on the screen. After the mouse click had been recorded, a 600-ms blank interval was presented before the next trial began.

In the motion block, the three visual as well as the three tactile stimuli implied motion, that is, the stimuli were presented adjacent to each other translating in a consistent direction in every trial. In the tactile modality, adjacent tactors vibrated successively, e.g., for a motion in the left-to-right direction (toward the wrist) which ended on tactor C, first tactor A, then tactor B, and lastly, tactor C vibrated (for the location of the tactors, see Fig. 1). Differences in directional sensitivity between left-to-right (toward the wrist) and right-to-left (toward the elbow) were not evidenced (see Merz, Meyerhoff, et al., 2019, pilot study). Tactors C (sequence: A – B – C) or D (sequence: B – C – D) were used as the last location for those trials indicating a motion in the left-to-right direction, tactors C (sequence: E – D – C) or B (sequence: D – C – B) were used as the last location for trials indicating a motion in the right-to-left direction. Please note that as the tactors were always attached to the left forearm, the left-to-right motion trials always approached the wrist, while the right-to-left motion trials always approached the elbow. For the visual modality, the vertical position (*y*-position) of the stimulus was fixed at the center of the upper half of the screen (at 300 pixels / ~8.1 cm from the upper edge of the screen). Given that the implied motion of the visual stimuli was along the horizontal axis, the latter also implied motion in the left-to-right / right-to-left direction. That is, the three stimuli in the motion block were presented with a center-to-center distance of 30 pixels / 0.81 cm (this corresponds to a visual angle of 0.77° at a viewing distance of 60 cm). Therefore, the speed of the visual stimulus was 1.62 cm/s (60 pixel/s), while the speed of the tactile stimulus was about 7 cm/s. The horizontal location of the last / third visual stimulus, as well as the only visual stimulus in the baseline block, was selected randomly, but restricted to be within ± 2.7 cm (± 100 pixels) of the center.

Overall, the participants completed 320 motion trials (160 visual target and 160 tactile target judgement trials). In the motion block, participants were given the chance for a break every 40 trials. In the motion block trials, the motion information was either congruent (i.e., in the same direction), or incongruent (i.e., in different directions). Both directions (left-to-right and right-to-left) were presented equally often. Finally, the tactile locations of the third vibration (tactors C and B for the right-to-left condition; tactors C and D for the left-to-right condition) were used equally often. For the baseline trials, the selection of the location of the visual and tactile stimuli were identical to the selection of the third location of the motion blocks.

#### Design and data-preparation

The participants were tested in a 2 × 2 × 2 design with the within-participants factors of *target modality* (visual vs. tactile), *directional congruency* (congruent vs. incongruent), and *target direction* (left-to-right vs. right-to-left). The factors *target modality* as well as *target direction* specify the modality / direction of the to-be-judged stimulus. As the dependent variable, we measured the horizontal localization error (i.e., the displacement along the *x*-axis). In a first step, the raw data were prepared for analysis, separately for each modality. All trials (baseline and motion trials combined) with a reaction time (RT) that was 1.5 interquartile ranges above or below the third quartile of each participant’s individual RT distribution (Tukey, 1977), were excluded from the data analysis. The RT criterion was used to exclude trials in which participants gave an atypical fast or slow response (e.g., due to tiredness, lack of focus/patience), as the timing between and within trials was fixed, with only the possibility for breaks every 40 trials in the motion block. Similarly, all trials with a location estimation (in pixels) along the horizontal (*x*-axis) or vertical (*y*-axis) axis that was 1.5 interquartile ranges above or below the third quartile of each participant’s individual location estimation distribution (Tukey, 1977), were excluded from the data analysis (see Steenbergen, Buitenweg, Trojan, & Veltink, 2014, for a similar approach). The data preparation of the location estimation was conducted for each location which served as the final location (visual modality: all final locations together; tactile modality: separate for the three final locations [B, C, and D]). Given these restrictions, 3.81% of the trials were excluded from the data analysis (tactile: 3.06%, visual: 4.55%)^1^.

In a second step, the localization error was computed. For the tactile modality responses, the mean location estimation of the motion trials was related to the mean location estimation of the baseline trials for each final location. For the localization error, a positive value indicates a displacement in the motion direction (e.g., Hubbard, 2005). For example, in a left-to-right (right-to-left) trial, a positive value would indicate a location estimation to the right (left) of the mean baseline estimation. Please note that it is important to relate the estimations of the motion trials to the estimation of the baseline trials, as there is no objectively correct location of the tactile stimulus due to the fact that the perceived location had to be indicated on a drawing of a forearm. As the size of the participants forearm and the size of the drawing were probably slightly different, participants need to transform the size of their forearm to the drawing shown on the screen. The baseline estimations provide a motion-free estimate about the location at which the participant indicated the final location on the drawing of the forearm. For the visual modality responses, each response was related to the actual final location of the stimulus. The localization errors were computed in a manner analogous to the tactile modality. For example, a positive localization error in a left-to-right (right-to-left) condition indicates a location estimation to the right (left) of the mean baseline estimation. This was done to enable comparability between the tactile and visual scores. All pixel values were transformed into mm scores, mean localization errors are displayed in Table 1. An analysis of the baseline trials is reported in the Appendix.

**Table 1.** Mean localization errors (in mm) as a function of target modality (visual vs. tactile), target direction (right-to-left vs. left-to-right), and directional congruency (congruent vs. incongruent) in Experiments 1 and 2. Standard deviations in parentheses

	Visual		Tactile	
	Right-to-left	Left-to-right	Right-to-left	Left-to-right
*Experiment 1*				
Congruent	6.80 (5.10)	4.87 (4.10)	– 10.58 (18.19)	3.90 (13.19)
Incongruent	6.49 (4.17)	5.29 (3.67)	– 16.13 (19.00)	– 3.85 (16.67)
*Experiment 2*				
Congruent	0.78 (1.80)	0.61 (1.91)	– 2.44 (16.58)	8.56 (16.48)
Incongruent	0.60 (1.76)	0.98 (1.94)	– 15.98 (15.80)	– 8.60 (21.04)

### Results

Visual motion information biased the localization of the tactile stimulus, but not *vice versa*, as shown by the 2 (target modality) × 2 (directional congruency) × 2 (target direction) analysis of variance (ANOVA). In general, localization errors differed as a function of the modalities, *F*(1, 21) = 57.18, *p* < .001, ɳ_p_² = .731, with a positive displacement being evidenced for the visual modality (5.9 mm), *t*(21) = 10.18, *p* < .001, *d* = 2.17, and a negative displacement for the tactile modality (– 6.7 mm), *t*(21) = – 5.07, *p* < .001, *d* = 1.08. Overall, the main effect of directional congruency was significant, *F*(1, 21) = 11.86, *p* = .002, ɳ_p_² = .361; but critically, the main effect interacted with target modality, *F*(1, 21) = 20.44, *p* < .001, ɳ_p_² = .493. Congruency had no effect in the visual modality, *t*(21) = – 0.12, *p* = .905, but was significant in the tactile modality, *t*(21) = – 4.02, *p* = .001, *d* = 0.86. These results therefore demonstrate that visual motion information biases perceived tactile location in the direction of the motion signal. In contrast, the direction of the tactile stimulus had no influence on the localization of the visual stimulus. Overall, the main effect of target direction was not significant, *F*(1, 21) = 3.31, *p* = .083, ɳ_p_² = .136, yet target direction interacted with target modality, *F*(1, 21) = 5.15, *p* = .034, ɳ_p_² = .197. Tactile localization errors were influenced by the direction of the tactile stimulus, *t*(21) = – 2.09, *p* = .049, *d* = 0.45, yet, the visual localization errors were independent of visual direction, *t*(21) = – 1.20, *p* = .243. The interaction between directional congruency and target direction, *F*(1, 21) = 0.31, *p* = .584, ɳ_p_² = .014, as well as the three-way interaction, *F*(1, 21) = 2.44, *p* = .133, ɳ_p_² = .104, were not significant.

### Discussion

The results of Experiment 1 reveal an influence of direction information on spatial localization of a different stimulus. More precisely, the irrelevant directional information in the visual modality influenced the spatial localization of the tactile stimulus, but not *vice versa*. Yet, the experimental set-up of Experiment 1 included systematic differences between the visual and tactile stimuli. The spatial separation between adjacent stimuli was different for the two modalities, as the center-to-center spacing in the tactile modality was approximately 3.5 cm, the spacing in the visual modality between adjacent stimuli was 30 pixels, corresponding to about 0.81 cm. Furthermore, in the visual modality, the participants indicated the actual location of the stimulus, whereas for the tactile modality, participants indicated (or referred to) the perceived location on a drawing of an arm on the computer screen. Therefore, the estimation of tactile location was much more indirect, as the actual location on the forearm had to be transferred on the generic arm presented on the computer screen.

In Experiment 2, a touch-tablet with five tactors attached to the back were used to present both the visual and tactile stimuli. This change in the design enabled us to present the visual and tactile stimuli from approximately the same locations (see Fig. 1) and participants tapped at the actually perceived location of the visual or tactile stimulus on the tablet.

## Experiment 2

### Method

#### Participants

Twenty-four new participants from the University of Trier took part in this study. The data from one participant was excluded due to their localization errors deviating more than 34 SDs from the group mean (5.54 cm, group mean = 0.07 cm, SD = 0.16 cm). All of the 23 remaining participants (two male, mean age, 20.96 years, 18–28 years old, two left-handed) reported normal or corrected-to-normal vision and no sensory impairment on their forearms.

#### Apparatus and stimuli

The apparatus and stimuli were identical to those used in Experiment 1 with the following exceptions. Five tactors were attached to the back of a 7” Touch monitor (faytech AG, Witzenhausen, Germany, resolution: 1680 x 1050 pixels; PPcm: 104.33). In between the tactors and the monitor, a piece of foam was placed in order to increase our participants’ comfort. Participants indicated the perceived location of the stimuli with the help of the touch stylus of the monitor. The central tactor was placed approximately at the center of the monitor, the other tactors were once again ordered in a straight line with an approximate center-to-center distance of 3.5 cm to the left and right of the central tactor (see Fig. 1). The blue visual stimulus (60 x 60 pixels; 0.57 x 0.57 cm) was presented on five different locations on the touch monitor, approximately spatially aligned with the tactors on the back of the tablet. Therefore, the speed of the visual and tactile stimulus was approximately identical at about 7 cm/s. The *y*-value of the visual stimulus was fixed for all five locations at 525 pixels, at the middle of the screen along the vertical axis. As the tactors were placed on the back of the tablet with an approximate center-to-center distance of 3.5 cm, the visual stimulus was presented with a center-to-center distance of 365 pixels to match the 3.5 cm center-to-center distance of the tactors.

#### Procedure

The procedure was identical to Experiment 1 with the following exceptions. After the presentation of the last vibration / visual stimulus, the background turned red (tactile) or green (visual) to indicate which modality was response relevant. The background color stayed red / green until a response was detected. No additional information was presented on the display. The participants indicated the perceived position with the touch stylus. The last locations used for left-to-right motion were locations C and D, the last locations used for right-to-left motion were locations C and B, in line with the locations used in Experiment 1. Once again, the touchscreen with the added tactors was attached to the left forearm of the participants, therefore all left-to-right motion trials (independent of modality) approached the wrist, while the right-to-left motion trials approached the elbow. The participants completed 320 motion trials. The number of baseline trials was adjusted to 72 baseline trials per block to balance the nine combinations of simultaneous presentation of the visual and tactile location (location B, C, and D for both modalities).

#### Design and data preparation

The participants were once again tested in a 2 × 2 × 2 experimental design with the within-participants factors of *target modality* (visual vs. tactile), *directional congruency* (congruent vs. incongruent), and *target direction* (left-to-right vs. right-to-left). For the tactile modality data, the data preparation was identical to Experiment 1. Comparable to the tactile data, only three distinct final locations had to be estimated in the visual modality. Therefore, the outlier analysis was conducted for each final location separately, identical to the tactile data preparation. Due to the data preparation, 10.98% of trials were excluded from data analysis (tactile: 9.25%, visual: 12.71%)^2^. To ensure comparability between experiments, the visual as well as tactile localization errors were computed in the same manner as in Experiment 1; that is, the motion trials were related to the mean location estimates of the baseline trials for each final location.

### Results

Replicating Experiment 1, the visual motion information influenced the localization of the tactile stimulus, but not vice versa (mean localization errors are depicted in Table 1). As for Experiment 1, localization errors for the last locations were different between the senses, *F*(1, 22) = 6.99, *p* = .015, ɳ_p_² = .241, a positive displacement was evidenced for the visual modality (0.74 mm), *t*(21) = – 2.26, *p* = .034, *d* = 0.47, while a negative displacement was observed in the tactile modality (– 4.62 mm), *t*(21) = – 2.31, *p* = .030, *d* = 0.48. Yet again, the main effect of directional congruency was significant, *F*(1, 22) = 11.50, *p* = .003, ɳ_p_² = .343; but critically, directional congruency interacted with target modality, *F*(1, 22) = 12.69, *p* = .002, ɳ_p_² = .366. As in Experiment 1, directional congruency had no effect in the visual modality, *t*(22) = – 0.50, *p* = .618, but did in the tactile modality, *t*(22) = 3.48, *p* = .002, *d* = 0.73. The pattern of results did not change, visual motion information biased perceived tactile location, but not vice versa. The main effect of target direction, *F*(1, 22) = 4.80, *p* = .039 ɳ_p_² = .179, and, comparable to Experiment 1, the interaction with modality, *F*(1, 22) = 4.95, *p* = .037, ɳ_p_² = .184, were significant. Once again, tactile localization errors were influenced by the direction of the tactile stimulus, *t*(22) = 2.21, *p* = .038, *d* = 0.46, but visual localization errors were unchanged by the direction of the visual stimulus, *t*(22) = 0.33, *p* = .745. The interaction between directional congruency and target direction, *F*(1, 22) = 2.31, *p* = .143, ɳ_p_² = .095, as well as the three way interaction, *F*(1, 22) = 2.94, *p* = .100, ɳ_p_² = .118, were, once again, not significant.

Due to the fact that we had to use partially different final locations for the different motion directions (location C was used for both motion directions, but location D only for left-to-right motions, and location B only for right-to-left motions), the congruent and incongruent condition did not just differ along the dimension of motion direction (same vs. different direction), but also along the dimension of final locations used. That is, the final location of the response irrelevant stimulus was either not shifted compared to the final location of the target stimulus, or it was shifted in or against the direction of motion of the response relevant target stimulus. Previous evidence has suggested that the location of a response irrelevant stimulus is able to influence the perceived location of a different stimulus in a visuotactile setting (called *spatial ventriloquism*, e.g., Pick et al., 1969; Shore, Barnes, & Spence, 2006; for an analysis of the spatial ventriloquism effect in the baseline trials, see the Appendix). To exclude this possible alternative explanation, we once again conducted a 2 (target modality) × 2 (directional congruency) × 2 (target direction) ANOVA, but only with those trials in which the tactile as well as visual final location was location C. The results did not change, exhibiting a significant interaction between target modality and directional congruency, *F*(1, 22) = 4.93, *p* = .037, ɳ_p_² = .183, as well as between target modality and target direction, *F*(1, 22) = 6.73, *p* = .017, ɳ_p_² = .234. Therefore, neither the directional difference between the two stimuli, nor any difference in their final locations, was responsible for the observed data pattern.

### Discussion

With the experimental set-up used in Experiment 2, we were able to eliminate many systematic differences between the estimation procedure of the visual and tactile stimulus. Furthermore, we also made the two motion stimuli more physically comparable by using similar locations from which the stimuli were presented. Yet, the results of Experiment 2 replicate those of Experiment 1. That is, an overall negative displacement for the tactile modality, and an overall positive displacement for the visual modality was obtained.^3^ As in Experiment 1, the influence of motion information in one modality on localization performance in the other sensory modality was asymmetrical between the two modalities. That is, the visual directional information influenced the localization of the tactile stimulus, but not *vice versa*. This influence of vision on touch might have been supported by the visual cues used to indicate the response relevant stimulus (e.g., Spence & Driver, 1996), yet, the usage of a modality neutral cues (e.g., a high or low-frequency auditory tone) was not feasible as the participants needed to wear earplugs as well as headphones with Brown noise playing to overshadow the sounds elicited by the tactors.

## General discussion

The present study was designed to fill the current gap in terms of studies in the crossmodal literature concerning the impact of motion information on spatial location. Across two experiments, we obtained a crossmodal influence of visual directional information on judgments of spatial location in the tactile modality. The spatial localization of the visual stimulus was not biased by the motion information of the tactile stimulus. In contrast, the localization of the tactile stimulus was biased by the direction of the motion information of the visual stimulus. Please note that our experimental setup and analyses were specifically designed so that this effect was an effect of motion information on localization, and that systematic difference between the final location of the response relevant target and response irrelevant stimulus have been accounted for (see the Results section of Experiment 2). This indicates a strong influence of the visual stimulus on the perceptual processing of the tactile stimulus.

Our observation of a unidirectional influence of visual motion information on tactile localization performance corresponds well to previous studies addressing visuotactile localization. As far as crossmodal influences in visuotactile localization (e.g., Pick et al., 1969; Shore et al., 2006), direction discrimination (e.g., Craig, 2006; Soto-Faraco et al., 2003), or congruency tasks are concerned (Spence & Walton, 2005; Walton & Spence, 2004), vision typically exhibits a much stronger influence over touch than vice versa. This is in line with the idea that our cognitive system creates a robust percept by minimizing perceptual uncertainty / variance (Ernst & Banks, 2002; Ernst & Bülthoff, 2004). Using vision to localize stimuli is much more accurate than using touch (especially when participants could use foveal vision, as in the present study). Therefore, the representation of visual information shows less variance than tactile / proprioceptive information (Ladwig, Sutter, & Müsseler, 2012, 2013; Pick et al., 1969; Sheth & Shimojo, 2001). As a result, vision is expected to mostly bias tactile / proprioceptive information, but not vice versa. In line with that, increasing the perceptual uncertainty about the visual stimulus would be expected to result in a crossmodal influence of touch on vision, comparable to evidence in different experimental settings (e.g., object height perception, Alais & Burr, 2004; Ernst & Banks, 2002).

The results of the present study are therefore consistent with classical crossmodal studies on the processing of stimulus location (spatial ventriloquism effect, e.g., Pick et al., 1969; Shore et al., 2006) or stimulus direction (crossmodal dynamic capture, e.g., Alais & Burr, 2004), but take them a step further. It is important to note that in these classical studies, both stimuli were manipulated along the same feature dimension (e.g., ‘direction’ [‘location’] for the crossmodal dynamic capture [spatial ventriloquism] literature) on which they were subsequently probed (once again, ‘direction’ [location]). We used this experimental logic, while subsequently not probing the perceived direction, but the perceived location. Still, we were able to evidence a systematic bias of visual motion on tactile localization, in line with the evidence of unimodal studies (representational momentum literature, e.g., Freyd & Finke, 1984) as well as crossmodal effects of these stimulus dimension in isolation. The current study thus joins and extends the existing literature on crossmodal correspondences (see Spence, 2011, for a review), which investigated correspondences between different dimension across modalities (e.g., visual spatial elevation with pitch, Deroy, Fernandez-Prieto, Navarra, & Spence, 2018; Melara & O'Brien, 1987).

For the general influence of motion on localization in the task-relevant modality, we documented a forward displacement in the visual modality in line with a large body of evidence on vision (Freyd & Finke, 1984; see Hubbard, 2005, 2018, for reviews). In the present study, participants did not know until after stimulus presentation which modality would be probed, therefore they had to divide their attention between the two modalities. This division of attention might be expected to enhance the crossmodal influence of visual directional information on tactile localization (although the way in which attention influences crossmodal / multisensory perception is currently debated, Odegaard, Wozny, & Shams, 2016). Yet, it is reasonable to assume that crossmodal influences still would have occurred when participants would be able to focus one stimulus, as crossmodal directional effects have been shown under such conditions (see Sanabria, Soto-Faraco, & Spence, 2007). Further, divided attention between two simultaneously-presented visual stimuli typically results in increased forward displacement (Hayes & Freyd, 2002, although, dividing attention between the senses is not necessarily the same as dividing attention within one sense, see Murphy, Dalton, & Spence, 2017). Yet, comparing the present results with other studies which have used stimuli with similar velocities (Exp 1: 1.62 cm/s; Exp 2: 7 cm/s) is difficult, as we presented a horizontal motion stimulus with only three stimuli, whereas, to the best of our knowledge, all studies investigating horizontal motion use at least five stimulus presentations or continuous motion (e.g., De Sá Teixeira, Hecht, & Oliveira, 2013; Hubbard & Bharucha, 1988; Hubbard & Ruppel, 2014).

Interestingly, for the tactile stimulus, we observed a backward displacement, in line with other tactile studies that used similar stimulus velocities (e.g., Macauda et al., 2018: 6 cm/s [slow condition]), yet in contrast to our own previous studies (Merz, Deller, et al., 2019; Merz, Meyerhoff, et al., 2019). This is surprising as central features of the task (stimulus duration, interstimulus interval, stimulus intensity, inter-tactor spacing, presentation of the stimuli at the left forearm) were similar between the present study and in our previously-published research which solely focused on the tactile modality (Merz, Deller, et al., 2019; Merz, Meyerhoff, et al., 2019). In fact, following the results of Hayes and Freyd (2002), we should have observed an increased forward shift under conditions of divided attention in the present study. Yet, one possible crucial difference between these studies is that baseline performance was assessed in separate blocks of experimental trials. That is, for the motion block, all 100% of the trials implied a motion in a specific direction. In contrast, in our previous studies, the baseline / control trials were mixed with motion trials within a single experimental block, therefore only half of the trials implied motion in a specific direction. This resulted in different trial contexts between these studies. Note that context has previously been shown to influence perceptual processing (e.g., Found & Müller, 1996; Wolfe, 1994), selection (e.g., Frings, Merz, & Hommel, 2019), and crucially, the localization of the onset of a moving stimulus (onset-repulsion effect / Fröhlich effect, see Müsseler & Tiggelbeck, 2013; for review, see Müsseler & Kerzel, 2018). In line with this idea, Macauda et al. (2018) only used motion trials in their study. Similarly, in one experiment of our study (Merz, Deller, et al., 2019, Experiment 2b), we also presented only implied motion trials. In this experiment, proximal (distal) trials with a motion direction toward the elbow (wrist), were perceived as closer to the wrist (elbow) for the central tactor location, in line with a backward displacement. Yet, without a systematic investigation in this topic, this must remain as mere speculation at this point.

## Conclusions

In the present visuotactile study, we observed a crossmodal influence of visual directional information on judgments of spatial location in the tactile modality. This emphasizes the close interplay between motion and localization, in line with unisensory evidence (see Hubbard, 2005, 2018). This influence was unidirectional, the direction of a visual stimulus influences perceived tactile location, but not vice versa. This result is in line with current theoretical frameworks which express the idea of incorporating information from the different senses in order to minimize perceptual uncertainty and perceptual variance in the final, multisensory percept (Ernst & Bülthoff, 2004).

## Figures and Tables

**Fig. 2.**
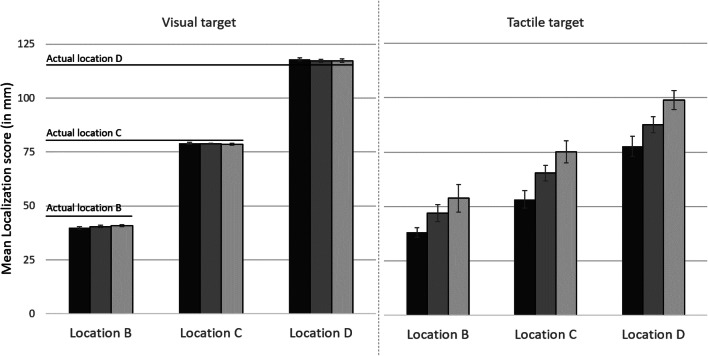
Mean localization score as a function for the visual target (*left side*) and tactile target (*right side*) baseline trials in Experiment 2. Mean localization scores are depicted as a function of target location (*x*-axis) and response irrelevant stimulus location (*black*: location B; *dark grey*: location C; *light grey*: location D). For the visual target, actual stimulus location is indicated. *Error bars* represent the standard errors of the mean

## Appendix

### Baseline trials—analysis of general localization biases and spatial ventriloquism

Here we report a focused analysis of the baseline data. The participants were presented with two stimuli simultaneously (one visual, one tactile), and then were cued which stimulus had to be estimated (for more details, see the Procedure section). In a first step, we analyzed general localization biases which might have occurred and which have been observed in vision (although mostly in the periphery, e.g., Sheth & Shimojo, 2001) as well as in touch (e.g., Brooks, Seizova-Cajic, & Taylor, 2019). In a next step, we test for the occurrence of spatial ventriloquism, that is, we analyzed if the location of the visual stimulus influenced the localization of the tactile stimulus, and vice versa. In line with the idea that foveal vision is much more precise than tactile perceptual processing on the forearm (e.g., Gallace & Spence, 2014; Wässle et al., 1990; Weinstein, 1968), we expected to find an influence of the visual stimulus location on the tactile stimulus localization, but not vice versa (Pick et al., 1969). Please note that the baseline trials were split into two blocks at the beginning and the end of the experiment to account for possible localization biases which might manifest themselves during the experiment. Yet, comparing the overall localization scores between the two blocks did not reveal any biases in Experiment 1 (visual: *t*(21) = 1.27, *p* = .219; tactile: *t*(21) = 1.05, *p* = .305) as well as Experiment 2 (visual: *t*(22) = 1.09, *p* = .287; tactile: *t*(22) = – 0.30, *p* = .767). Therefore, the baseline estimations were collapsed across blocks to compute the localization error scores, reported in the main text, as well as the analyses reported in this appendix.

#### Experiment 1

For the visual stimulus, the indicated location during baseline trials did not differ from the actual location of the stimulus (mean difference: 0.09 mm), *t*(21) = 0.13, *p* = .896. Furthermore, as expected, the visual localization performance was not influenced by the location of the tactile stimulus (three possible locations: tactor location B, C, and D), *F*(2, 42) = 0.31, *p* = .732, ɳ_p_² = .015. This shows that no spatial ventriloquism has occurred from touch on vision. To indicate the perceived final location of the tactile stimulus, participants had to transform the actual stimulus location on their own forearm onto a drawing on the computer screen. The actual arm length of the participant, together with the exact tactor location on the forearm, was not recorded, therefore, no objectively correct estimation exists. As such, no localization bias can be estimate for the tactile stimulus.

#### Experiment 2

Three different locations were probed for the visual as well as tactile stimulus (locations B, C, and D). The center of the visual stimulus was located at 475 pixel (45.5 mm from the start of the display on the left; location B), 840 pixel (80.5 mm, location C), and 1205 pixel (115.5 mm, location D), and the locations of the tactors on the back were approximately matched. In a first step, general localization biases were tested in the visual modality. The distance between left location B and right location D is perceived to be greater than it actually is (actual distance: 70 mm; perceived distance: 77.07 mm), *t*(22) = 7.83, *p* < .001, *d* = 1.63, and we are not sure why this perceived extension of space occurred. More precisely, for all three locations, localization biases were found (for an illustration, see Fig. 2), that is, the right location (D) was located even further to the right (1.98 mm), *t*(22) = 4.16, *p* < .001, *d* = 0.87, whereas for the central (– 1.70 mm, location C), *t*(22) = – 5.20, *p* < .001, *d* = 1.08, and the left (–5.12 mm, location B), *t*(22) = – 7.42, *p* < .001, *d* = 1.55, location, participants estimates were located to the left of the actual stimulus location. For the tactile stimulus, the reverse data pattern was found, that is, the distance between the left location B and the right location D was perceived to be smaller than it actually is (actual distance: 70 mm; perceived distance: 41.74 mm), *t*(22) = –5.56, *p* < .001, *d* = 1.16. This contraction of space is in accordance with several findings in the tactile modality that stimuli are perceived toward the center of stimulation (e.g., Brooks et al., 2019). As the actual tactor location cannot be matched with absolute certainty to a specific location on the screen, localization biases for each location were not computed.

In a second step, crossmodal influences between vision and touch were tested. The usage of three distinct locations for both modalities resulted in nine stimulus combinations. A 2 (*target modality:* visual vs. tactile) x 3 (*target location*: location B vs. location C vs. location D) x 3 (*response irrelevant stimulus location:* location B vs. location C vs. location D) ANOVA was conducted using absolute location estimation scores as dependent variable (higher scores indicate a localization to the right, see also Fig. 2). If the sphericity assumption was violated, Greenhouse–Geisser corrections were performed. Of course, from the left (location B) to the right location (location D), the localization scores increased, as indicated by the main effect of target location, *F*(1.40, 30.75) = 423.94, *p* < .001, ɳ_p_² = .951. The main effect of target modality was significant, *F*(1, 22) = 18.76, *p* < .001, ɳ_p_² = .460, indicating a localization closer to the left for tactile stimuli (66.36 mm) than for visual stimuli (78.90 mm). Target location and target modality interacted, *F*(1.27, 28.00) = 38.85, *p* < .001, ɳ_p_² = .638, confirming the result from the first analysis that the perceived distance in the visual modality was greater than in the tactile modality. The main effect of response irrelevant stimulus location was significant, *F*(1.06, 23.31) = 15.73, *p* < .001, ɳ_p_² = .417, once again indicating an increase in localization scores from the left (location B) to the right (location D). Yet, most importantly, only the visual response irrelevant stimulus location had an influence on the tactile location, but not vice versa, as indicated by the significant interaction with target modality, *F*(1.06, 23.41) = 16.75, *p* < .001, ɳ_p_² = .423. This result is in line with our predictions and the results from previous studies (e.g., Pick et al., 1969).

## Footnotes

1 The 2 x 2 x 2 ANOVA was also conducted without the exclusion of any trials. The same pattern of results was obtained as reported in the main text.

2 Once again, the 2 x 2 x 2 ANOVA, reported in the results section, was also conducted without the exclusion of any trials. The pattern of results were mostly the same, only the main effect of target direction (*p* = .069) and the interaction between target modality and target direction (*p* = .118) did not reach significance, while the three-way interaction did (*p* = .012). Yet, most importantly, the results with and without the exclusion of trials were exactly the same for the analysis only focusing on trials with tactor C as the final location.

3 The effect sizes for the positive displacement in the visual modality differed between Experiments (Experiment 1: *d* = 2.17; Experiment 2: *d* = 0.47). Several differences between the experimental set-ups like stimulus size (Exp 1: 0.81 x 0.81 cm; Exp 2: 0.57 x 0.57 cm), the distance between successive stimulus presentations (Exp 1: 0.81 cm; Exp 2: 3.5 cm) and therefore also stimulus speed (Exp 1: 1,62 cm/s; Exp 2: 7 cm/s), and possible changes in background colour at final location from stimulus presentation (no changes; Exp 1 & 2: dark grey) to response collection (Exp 1: still dark grey; Exp 2: green) possibly have added uncertainty and therefore variance to the participant’s estimate in Experiment 2, resulting in a decrease of effect size from Experiment 1 to 2.
